# A machine learning-derived genomic dataset from bacteria frequently reported as probiotics

**DOI:** 10.3389/fbinf.2026.1810235

**Published:** 2026-04-22

**Authors:** Diego Lucas Neres Rodrigues, Pedro Alexandre Sodrzeieski, Sandrine Auger, Jean-Marc Chatel, Ana Maria Benko-Iseppon, Vasco Azevedo, Siomar de Castro Soares, Flávia Figueira Aburjaile

**Affiliations:** 1 Federal University of Minas Gerais, Belo Horizonte, Minas Gerais, Brazil; 2 Federal University of Pernambuco, Recife, Pernambuco, Brazil; 3 MICALIS Institute, INRAe, Jouy-en-Josas, France; 4 Federal University of Triângulo Mineiro, Uberaba, Minas Gerais, Brazil

**Keywords:** bioinformatics, data mining, data science, gut microbiota, probiogenomics

## Abstract

Probiotics are live microorganisms that have been widely investigated for their association with beneficial host outcomes, particularly in the context of gut-associated microbial communities. Despite extensive literature, the probiotic effects are recognized as strain-specific and highly context-dependent, which limits the identification of universal genetic determinants of probiosis. In this study, we present a machine learning-derived genomic dataset generated from comparative analyses of bacterial genomes belonging to taxa frequently reported as probiotics and reference gut-associated bacteria. Using pangenomic analysis combined with supervised machine learning approaches, including Random Forest, Support Vector Machine, and Logistic Regression, we extracted discriminative genomic features from large-scale genome data. The resulting dataset comprises 1,072 non-redundant protein-coding sequences, accompanied by gene presence-absence matrices and functional annotations. These features should not be interpreted as causal determinants of probiotic functionality, but rather as genomic patterns associated with bacterial taxa commonly used as probiotics, which may also reflect taxonomic and ecological signatures. All data and scripts used in this study are publicly available through an open-access repository, providing a reusable resource for exploratory analyses, comparative genomics, and methodological benchmarking in probiogenomics and microbial genomics. The final data, hereby called ProbioSML, is currently available on https://doi.org/10.5281/zenodo.14181443.

## 1 Introduction

Probiotic concepts were first introduced at the beginning of the 20th century through studies associating the consumption of fermented dairy products with increased longevity and improved wellbeing (Martín and Langella, 2019). Since then, scientific and technological advances have substantially expanded the scope of probiotic research, leading to the currently accepted definition of probiotics as live microorganisms that, when administered in adequate amounts, confer health benefits to the host (Kechagia et al., 2013; Zhang et al., 2022). This broad definition has driven extensive investigation into microorganisms commonly associated with human and animal health, particularly within the context of the gut microbiota.

From a microbiological and genomic perspective, the beneficial effects attributed to probiotic microorganisms are understood to arise from complex and context-dependent molecular interactions involving the host, the microbiota, and environmental factors (Lebeer et al., 2008). Several functional categories, including stress response, adhesion, and host-microbe interaction, have been recurrently reported in studies of probiotic strains (Lebeer et al., 2008; de Jesus et al., 2021; Ariute et al., 2023). However, these features are neither universal nor sufficient to define probiosis, as probiotic effects are widely recognized as strain-specific and highly dependent on host physiology and ecological context (Blandino et al., 2008; de Jesus et al., 2021). Consequently, no consensus exists regarding a universal genetic signature that defines probiotic functionality.

Despite these limitations, the identification and comparative analysis of genomic features present in microorganisms frequently associated with probiotic use remain highly relevant. Such analyses contribute not only to probiogenomics but also to broader questions in microbial ecology and general microbiology, particularly regarding bacterial adaptation, survival, and persistence within host-associated environments (O′Toole and Cooney, 2008). The availability of genome-scale datasets capturing these features enables the exploration of bacterial capacities related to colonization, competition, and resilience within complex microbial communities, which directly influence microbiota dynamics and host-microbe interactions (Chandrasekaran et al., 2024). These aspects highlight the broader implications of probiotic-associated research for public, environmental, and One Health perspectives.

The efficacy of specific microbial strains frequently reported as probiotics has been demonstrated in numerous clinical and experimental studies. A prominent example is *Lacticaseibacillus rhamnosus* GG, which has been extensively investigated for its association with reduced incidence of antibiotic-associated diarrhea (Waitzberg et al., 2024), prevention of respiratory tract infections (Liu et al., 2013), mitigation of atherosclerosis in murine models (Zhai et al., 2022), and antagonism against *Salmonella* infections (Capurso, 2019). Similarly, in animal health contexts, microorganisms such as *Enterococcus faecium* have been applied as feed or water additives in aquaculture, where they have been associated with improved growth performance and partial antagonism against pathogens including *Francisella orientalis* and *Streptococcus agalactiae* (Suphoronski et al., 2021). Comparable applications have been reported across a wide range of animal production systems, including cattle (Nalla et al., 2022; Saha et al., 2023; Wang et al., 2023), goats (Nalla et al., 2022), horses (Schoster, 2018), companion animals (Yang and Wu, 2023), and poultry (Khan and Naz, 2013).

These examples illustrate why certain bacterial taxa have been recurrently investigated and reported as probiotics across diverse hosts and environments. Importantly, however, such well-characterized cases should not be interpreted as evidence of a shared or universal genetic basis of probiosis. Instead, they underscore the historical and practical interest in particular taxonomic groups whose members have repeatedly demonstrated beneficial host associations under specific conditions.

Several public databases have been developed to organize probiotic-related information, including ProBioQuest (Chan et al., 2022), IPDB (Tarracchini et al., 2022), PROBIODB (Tao et al., 2017), and Probio-Ichnos (Tsifintaris et al., 2024). While these resources provide valuable insights derived from literature surveys, *in vitro* experiments, or curated strain collections, many are currently unavailable, limited in scope, or not designed to support large-scale comparative genomic or machine learning-based analyses. Notably, there remains a shortage of reusable, genome-scale datasets that systematically catalog genomic features of commonly reported probiotic bacteria, together with appropriate reference groups.

The increasing availability of large genomic datasets has highlighted the utility of machine learning approaches, such as support vector machines (SVM), logistic regression (LR), and random forest (RF), for exploratory data analysis in high-dimensional biological contexts (Greene et al., 2016). These methods are well-suited for identifying discriminative patterns within complex datasets and have been successfully applied to classification and feature selection tasks in microbial genomics. Importantly, in this context, machine learning approaches are employed as exploratory and descriptive tools, capable of generating datasets that reflect genomic patterns without implying causal relationships between individual features and complex phenotypes such as probiosis.

Considering these aspects, the development of a machine learning-derived genomic dataset represents a relevant contribution to probiogenomics and microbial genomics. Rather than attempting to define a universal genetic basis for probiotic effects, such a dataset so-called ProbioSML, might capture discriminative genomic features associated with bacterial taxa frequently reported as probiotics, which may also reflect taxonomic and ecological signatures. These data can subsequently support comparative analyses, methodological benchmarking, and hypothesis generation for future experimental studies. Accordingly, this work should be interpreted as a data resource rather than a functional characterization study.

## 2 Methods

**FIGURE 1:**
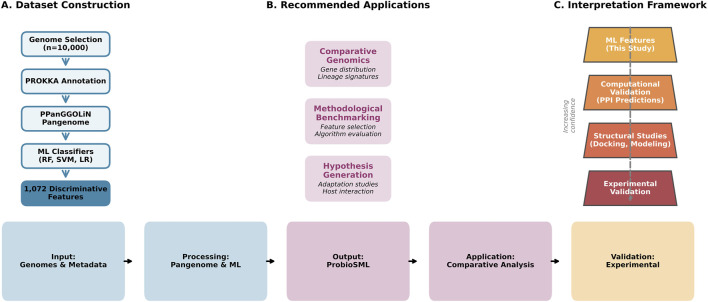
Overview of ProbioSML construction, applications, and interpretation framework. **(A)** Computational pipeline for dataset generation. **(B)** Recommended downstream applications with example use cases. **(C)** Hierarchical validation framework emphasizing the distinction between discriminative patterns present in this study and functional validation.

The methodological workflow employed in this study was designed to generate a reproducible, machine learning-derived genomic dataset through comparative analyses of bacterial genomes (Figure 1). The focus of the pipeline is the identification of genomic features that discriminate between genome groups, rather than the inference of causal relationships with probiotic phenotypes. All in-house scripts developed to perform data processing, statistical analyses, and feature extraction are publicly available through the Integrative Bioinformatics Laboratory GitHub repository (Rodrigues and Aburjaile, 2024).

### 2.1 Genome selection and data acquisition

Prior to data mining, bacterial genera were selected based on their frequent reporting in the literature as probiotics or probiotic candidates, as well as their relevance within gut-associated microbial communities. A complementary group of reference genomes was assembled from gut-associated bacteria not primarily described as probiotics. This grouping strategy was adopted to enable comparative analyses at the genome level and does not imply that all members of a given taxon exhibit probiotic properties.

Genome metadata for all selected taxa were retrieved from the National Center for Biotechnology Information (NCBI) repository, including both complete and draft genomes publicly available up to 22 July 2024. An automatic screening step was applied to exclude anomalous or incomplete assemblies. To minimize sampling bias and ensure computational feasibility, an in-house Python three script (Rodrigues and Aburjaile, 2024) was used to randomly select 5,000 genomes from each genome group. The selected 10,000 genomes were subsequently downloaded using the genome_picker.py script.

### 2.2 Genome annotation and pangenomic analysis

All selected genomes were automatically annotated using the PROKKA pipeline (Seemann, 2014), employing parameters optimized for rapid structural annotation. Following annotation, genomic data were reorganized into standardized directory structures separating genome sequences, predicted proteomes, and gene sets.

Pangenomic analysis was performed using the PPanGGOLiN tool (Gautreau et al., 2020), which enables the construction of a partitioned pangenome and the generation of a sparse binary matrix representing gene presence and absence across genomes. Identity and coverage thresholds were both set to 80% to define gene clusters. The resulting matrix served as the primary input for subsequent dimensionality reduction and machine learning analyses.

### 2.3 Dimensionality reduction and exploratory data analysis

To explore the structure of the high-dimensional pangenomic matrix and assess variable behavior, dimensionality reduction was performed using Principal Component Analysis (PCA). A cumulative variance plot was generated to determine the minimum number of principal components required to recover 90% of the total variance in the dataset. Additionally, two-dimensional projections based on the most explanatory components were visualized to inspect clustering tendencies among genome groups.

For exploratory clustering, the k-nearest neighbor (KNN) method was applied assuming k = 1. The value of k was determined using the elbow method (Shi et al., 2021). This step was performed solely for data visualization and exploratory assessment and was not used to infer biological relationships or functional causality.

### 2.4 Machine learning classifiers and feature extraction

To identify genomic features that discriminate between the predefined genome groups, three supervised machine learning classifiers were employed: logistic regression (LR), support vector machines (SVM), and random forest (RF). These methods were selected due to their widespread use in high-dimensional biological datasets and their complementary approaches to feature weighting and selection.

All classifiers were implemented using the scikit-learn library and integrated into a unified analysis pipeline available in the generateDB.py script. The sparse binary pangenomic matrix was used as the input for all models. Genome labels were assigned based on taxonomic grouping derived from literature reports, and these labels were used exclusively for comparative classification purposes.

For the LR model, 300 internal iterations were performed using an L2 regularization penalty to reduce overfitting, with the limited-memory Broyden-Fletcher-Goldfarb-Shanno (LBFGS) solver applied to handle the large feature space. The linear SVM model was configured with 100 external iterations, employing stochastic gradient descent (SGD) optimization and an L2 penalty, a configuration suitable for large-scale binary classification tasks. For the RF model, 100 external iterations were conducted using 100 estimators per iteration, with the Gini index applied as the impurity criterion.

Feature importance was extracted independently for each model: regression coefficients for LR, feature importance scores for RF, and weight vectors for SVM. Features were considered distinct if they appeared in at least 40% of the external iterations for RF and SVM. For LR, features were retained when their cumulative contribution exceeded 0.3% of the total dataset value. Importantly, selected features are interpreted as discriminative variables rather than as determinants of probiotic functionality.

### 2.5 Model evaluation

Model performance was evaluated using multiple accuracy metrics, including the area under the receiver operating characteristic curve (ROC AUC), F1-score, and k-fold cross-validation accuracy. For each classifier, the dataset was randomly shuffled and partitioned 100 times, with 90% of the data used for training and 10% reserved for testing in each iteration. Final performance values correspond to the average results across all iterations. These metrics were used to assess classification consistency and robustness rather than biological validity.

### 2.6 Functional annotation of discriminative features

To provide functional context for the extracted discriminative features, protein sequences corresponding to selected genes were functionally annotated using the egg-NOG-mapper pipeline (Cantalapiedra et al., 2021). Annotations were assigned based on orthology and mapped to Clusters of Orthologous Groups (COG) categories (Tatusov et al., 2000; 2003). A maximum e-value threshold of 6e-5 was applied during the mapping process. Functional distributions were summarized and visualized to facilitate comparative interpretation among feature subsets derived from different classifiers.

## 3 Results

### 3.1 Genome selection and dataset standardization

The bacterial species selected for the initial stages of the analysis, as well as the number of genomes available for each taxon up to the defined cutoff date, are provided in Supplementary Table S1. Detailed metadata for all genomes included in the final analyses are described in Supplementary Table S2. Due to the balance requirements imposed by supervised machine learning approaches, species-level groupings were adopted in-stead of strain-level classifications. This strategy enabled the construction of a computationally tractable dataset while preserving taxonomic representativeness across genome groups.

All selected genomes were successfully annotated and standardized, resulting in a uniform dataset suitable for downstream pangenomic and machine learning analyses. This preprocessing step ensured consistency in gene prediction and annotation across all genomes included in the study.

### 3.2 Dimensionality reduction and exploratory analysis

Principal Component Analysis (PCA) was applied to the sparse pangenomic matrix to explore the structure of the high-dimensional dataset. Supplementary Figure S1 illustrates the distribution of genomes projected onto the two principal components that explain the greatest proportion of variance. The projection reveals heterogeneity within the reference genome group, while genomes from taxa frequently reported as probiotics display a comparatively more compact distribution.

The application of the k-nearest neighbor (KNN) clustering method further highlights grouping tendencies within the reduced feature space. Although some degree of overlap between genome groups is observed, the clustering pattern suggests that the pangenomic features captured by the dataset encode sufficient information to discriminate between genome groups at a global level. This analysis was conducted for exploratory purposes only and does not imply functional or phenotypic differentiation.

### 3.3 Overview of the machine learning-derived dataset

Application of the three machine learning classifiers resulted in the extraction of distinct sets of discriminative genomic features. Logistic regression identified 1,047 genes, while random forest and support vector machine models selected 113 and 85 genes, respectively. After removal of redundant entries, the final non-redundant dataset comprises 1,072 protein-coding sequences. Among these, 524 sequences were annotated as hypothetical proteins.

**FIGURE 2:**
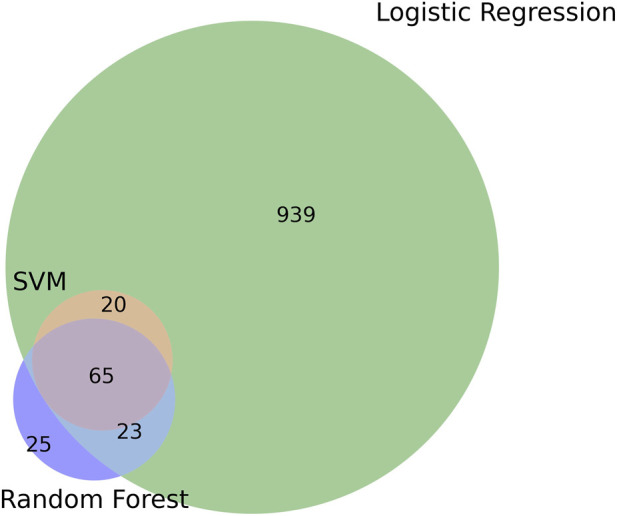
Venn diagram representing the total number of genes found by each method applied. Green represents the LR method. Purple represents the RF method. Red represents the SVM method.

The intersection of the three classification methods contains 65 genes, representing features consistently identified as discriminative across different modeling approaches (Figure 2). A substantial proportion of these intersecting features corresponds to orthologs originally identified in well-characterized strains such as *Escherichia coli* Nissle 1917 and *Enterococcus faecium* Symbioflor 1. Specifically, 43 proteins in the intersection set are orthologs from *E. coli* Nissle 1917, and three originate from *E. faecium* Symbioflor 1, accounting for approximately 70% of the intersection subset. In contrast, these strains contribute approximately 5.4% of the features when considering the full union of all selected genes.

This distribution reflects the influence of extensively studied taxa within the dataset and does not imply exclusivity of these features to specific strains. Instead, it highlights the recurrence of certain genomic elements within taxa that are frequently used as references in probiotic research.

### 3.4 Functional annotation of discriminative features

**FIGURE 3:**
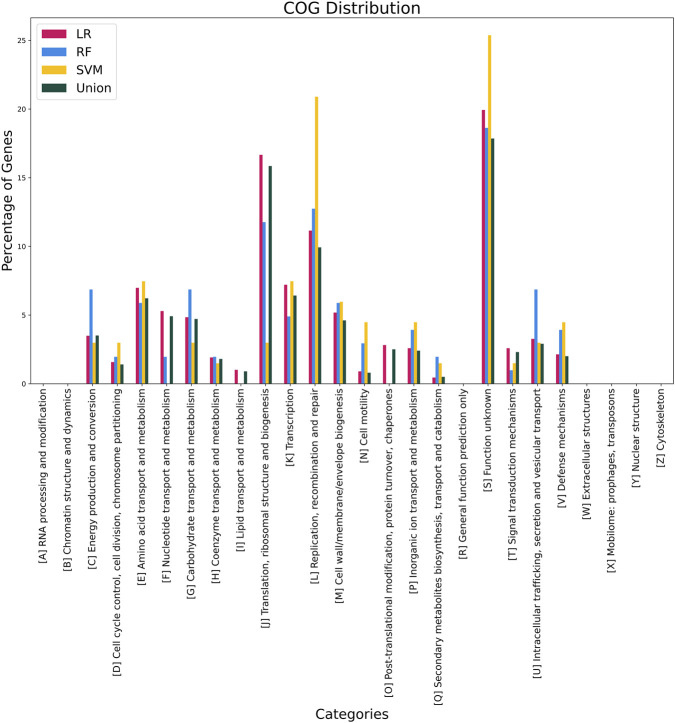
Bar graph that represents the distribution of found elements on each COG category by individual method. Methods: LR - Logistic regression; RF - Random Forest; SVM - Support vector machines; Union - Set representing the union of all previously mentioned methods.

Functional annotation using the eggNOG-mapper pipeline successfully assigned COG categories to 997 proteins from the non-redundant dataset. The distribution of functional categories reveals a predominance of genes associated with category S (function unknown; 17.85%), followed by category J (translation, ribosomal structure, and biogenesis; 15.85%) and category L (replication, recombination, and repair; 9.93%). The functional distribution of annotated proteins across classification methods and their union is illustrated in Figure 3.

The prevalence of proteins with unknown or general functions is consistent with exploratory, genome-scale analyses and reflects the current limitations of functional annotation in microbial genomics. The presence of genes related to core cellular processes indicates that discriminative features may capture patterns linked to bacterial growth, genome maintenance, and adaptation, which are common to taxa frequently reported as probiotics.

### 3.5 Performance evaluation of machine learning approaches

All machine learning models achieved high performance across multiple evaluation metrics (see Table 1). Average ROC AUC, F1-score, and cross-validation accuracy values exceeded 0.9 for all classifiers, with logistic regression and random forest models approaching near-perfect scores. These results demonstrate the robustness and consistency of the classification pipeline in discriminating between the predefined genome groups.

**TABLE 1:** Accuracy tests results obtained after 100 iterations.

Method	ROC AUC	F1-score	Cross-validation	Accuracy
Support vector machine	0.9794	0.9777	0.9794	0.9785
Logistic regression	0.9997	0.9997	0.9997	0.9995
Random forest	0.9998	0.9998	0.9998	0.9995

Importantly, high classification accuracy indicates strong separability of the genome groups within the feature space defined by the pangenomic matrix. These performance metrics reflect the discriminative capacity of the dataset and the modeling strategy rather than the biological validation of probiotic function.

### 3.6 Data usage

ProbioSML is designed as a reusable resource for three complementary applications: (I) comparative genomics, enabling the exploration of gene distribution patterns across gut-associated bacteria and the identification of lineage-specific genomic signatures; (II) methodological benchmarking, supporting the evaluation of feature selection algorithms and classification workflows in pangenomic contexts; and (III) hypothesis generation, facilitating the prioritization of candidate genes for experimental studies on bacterial adaptation, host interaction, and ecological persistence.

Users should download the FASTA file containing 1,072 protein-coding sequences and associated functional annotations from the Zenodo repository. For comparative analyses, we recommend employing local alignment tools to query ProbioSML sequences against target genomes, defining gene presence based on sequence similarity thresholds (≥80% identity and coverage, consistent with our pangenome construction parameters) or orthology inference. Following the presence-absence matrix construction, results should be interpreted considering the biological function of predicted proteins, their taxonomic distribution across bacterial lineages, and the ecological context of target strains. The users should also consider using the intersection set as a highly reliable source of data for probiotic-related taxa.

Features identified through machine learning reflect composite signals encompassing taxonomic relatedness, ecological adaptation, and historical research bias, but not universal determinants of probiotic function. Users integrating these data with phenotypic information must account for strain-specific context and host variables. The intersection set - 65 genes consistently identified across all three classifiers - offers higher-confidence candidates for follow-up studies; however, even these require experimental and mechanistic validation before functional claims can be made.

## 4 Discussion

Probiotic-associated microorganisms represent an important area of investigation within the One Health framework, given their reported relevance to both human and animal health (Harutyunyan et al., 2022; Tufail and Schmitz, 2024). Numerous studies have documented beneficial outcomes associated with the use of microorganisms commonly described as probiotics, including improved clinical outcomes and enhanced performance indicators in animal production systems, such as weight gain and survival rates (Piewngam et al., 2018; Schmitz, 2021; Ribera et al., 2024). Despite these advances, a persistent gap remains in the mechanistic understanding of probiotic-associated effects, which continues to limit the identification of novel molecules and the rational exploration of existing strains.

Considering the complexity and multidimensionality of this research field, careful construction of the initial genomic dataset was essential to ensure analytical consistency and reliability. For this reason, the present study was restricted to bacteria associated with the enteric microbiota, a system that has been extensively investigated and serves as a well-established model for host-microbe interactions (Wang et al., 2022; Han et al., 2024; Wong and Good, 2024). The bacterial taxa included were selected based on literature highlighting their relevance to gut microbial communities and their frequent association with beneficial host-related outcomes (Holzapfel et al., 2001; Lukjancenko et al., 2011; Abouelela and Helmy, 2024; Al-Fakhrany and Elekhnawy, 2024). Importantly, these selections reflect patterns of scientific reporting and historical research focus rather than assertions of universal probiotic functionality across all members of each taxon.

Machine learning approaches, particularly those incorporating feature selection strategies, have been increasingly applied to biological data for information retrieval and the construction of specialized datasets (Ishwaran et al., 2010; Li et al., 2017; Ho et al., 2019; Morais-Rodrigues et al., 2020). These methods enable scalable analysis of high-dimensional data while reducing noise and redundancy. In the present study, dimensionality reduction and clustering analyses revealed a notable degree of homogeneity within genome groups, as illustrated in Supplementary Figure S1. This observation suggests that the variables derived from the pangenomic matrix capture structured patterns within the data, supporting their explainability and consistency (Gerbrands, 1981; Zang et al., 2024). The high classification scores obtained across all models, with accuracy values exceeding 0.97, indicate that the selected variables are sufficient to discriminate between the predefined genome groups. These results reflect the internal coherence of the dataset and the effectiveness of the classification framework, rather than direct biological validation of probiotic properties.

The relatively limited overlap among genes selected by the three machine learning algorithms can be attributed to the distinct criteria each method employs to rank and prioritize features. Logistic regression, support vector machines, and random forest models emphasize different aspects of the data structure, leading to the identification of complementary but non-identical feature sets (Statnikov et al., 2008; Lin et al., 2018). This divergence is further amplified by the high dimensionality of the pangenomic matrix, in which multiple subsets of genes can yield similarly strong classification performance. Consequently, the reduced intersection among selected genes reflects methodological diversity and data complexity rather than inconsistency or lack of robustness.

Functional categorization of the extracted features revealed a predominance of genes assigned to COG category S, corresponding to proteins of unknown function. This outcome is characteristic of exploratory, genome-scale analyses and reflects the current limitations of functional annotation in microbial genomics. Uncharacterized proteins are unlikely to be retained in bacterial genomes without contributing to fitness or essential cellular processes, suggesting that they may play roles that remain to be elucidated (Wegrzyn, 2001; Berg and Kurland, 2002). Accordingly, the dataset provides opportunities for future investigations aimed at characterizing these proteins and exploring their potential biological relevance (Goodacre et al., 2013).

The presence of genes associated with core cellular functions, such as replication, recombination, repair, and translation, was also observed among the discriminative features. Rather than indicating direct probiotic mechanisms, these patterns may reflect broader differences in growth dynamics, genome maintenance, and ecological adaptation among gut-associated bacteria. Previous studies have reported that such functions are relevant to bacterial persistence, competition, and interaction with host environments (Lebeer et al., 2008; Yan and Polk, 2020; Chandrasekaran et al., 2024). In particular, work by Lebeer, Vanderleyden, and Keersmaecker emphasized the importance of DNA repair, stress response, and cell wall-related processes in strains commonly described as probiotics (Lebeer et al., 2008). These functions are intrinsically linked to bacterial survival, adaptability, and sustained colonization, as well as to mechanisms such as site competition and epithelial association (Saad, 2006; Lebeer et al., 2008). Consistent with these reports, all machine learning approaches employed in this study identified a substantial number of genes related to cell wall biosynthesis and maintenance, reinforcing the relevance of these pathways within the dataset.

Taken together, the results indicate that the assembled dataset is of satisfactory quality and suitable for reuse in a range of prospective analyses. The dataset captures genomic patterns associated with bacterial taxa frequently reported as probiotics, while also reflecting taxonomic structure, pangenomic diversity, and research biases inherent to publicly available genomic resources. Although the intersection set contains a high proportion of proteins originating from well-characterized probiotic strains, further experimental, structural, and ecological studies are required to advance the understanding of mechanisms underlying probiosis.

## 5 Conclusion

In this study, we present a machine learning-derived genomic dataset generated from comparative analyses of bacterial genomes belonging to taxa frequently reported as probiotics and reference gut-associated bacteria. Rather than attempting to define genetic determinants of probiotic functionality, the dataset captures discriminative genomic patterns that reflect taxonomic, ecological, and pangenomic structure.

By integrating pangenomic analysis with multiple supervised machine learning approaches, we provide a reproducible pipeline for feature extraction from large-scale genomic data. The resulting dataset is intended as a reusable resource for exploratory analyses, methodological benchmarking, and hypothesis generation in probiogenomics and microbial genomics.

Importantly, the genomic features identified here should not be interpreted as causal or definitive markers of probiosis. Probiotic effects remain strain-specific and context-dependent, requiring experimental and clinical validation. Within these limitations, the dataset contributes to ongoing efforts to organize and analyze genomic information associated with bacteria commonly investigated in probiotic research, supporting future studies at the interface of data science, microbiology, and One Health.

The final dataset and its metadata are available on https://doi.org/10.5281/zenodo.14181443.

## Data Availability

The datasets presented in this study can be found in online repositories. The names of the repository/repositories and accession number(s) can be found in the article/Supplementary Material.
