# Supplementary material for: A machine learning-derived genomic dataset from bacteria frequently reported as probiotics
Source: Front Bioinform. 2026 Apr 22;6:1810235. doi: 10.3389/fbinf.2026.1810235 (PMC13144117; doi:10.3389/fbinf.2026.1810235)
Supplement: Supplementary file 2 [file DataSheet1.pdf]

Supplementary Material

A machine learning-derived genomic dataset from bacteria frequently reported as probiotics

Diego Lucas Neres Rodrigues [1], Pedro Alexandre Sodrzeieski [1,2], Sandrine Auger [3], Jean-Marc Chatel [3], Ana Maria Benko-Iseppon [2], Vasco Azevedo [1], Siomar de Castro Soares [4] and Flávia Figueira Aburjaile [1,*]

[1]Federal University of Minas Gerais, Belo Horizonte, Minas Gerais, Brazil, 31270-901

[2]Federal University of Pernambuco, Recife, Pernambuco, Brazil, 50670-901

[3]MICALIS Institute, INRAe, Jouy-en-Josas, France, 78352

[4]Federal University of Triângulo Mineiro, Uberaba, Minas Gerais, Brazil, 38025-180

*** Correspondence:**
Corresponding Author
faburjaile@gmail.com

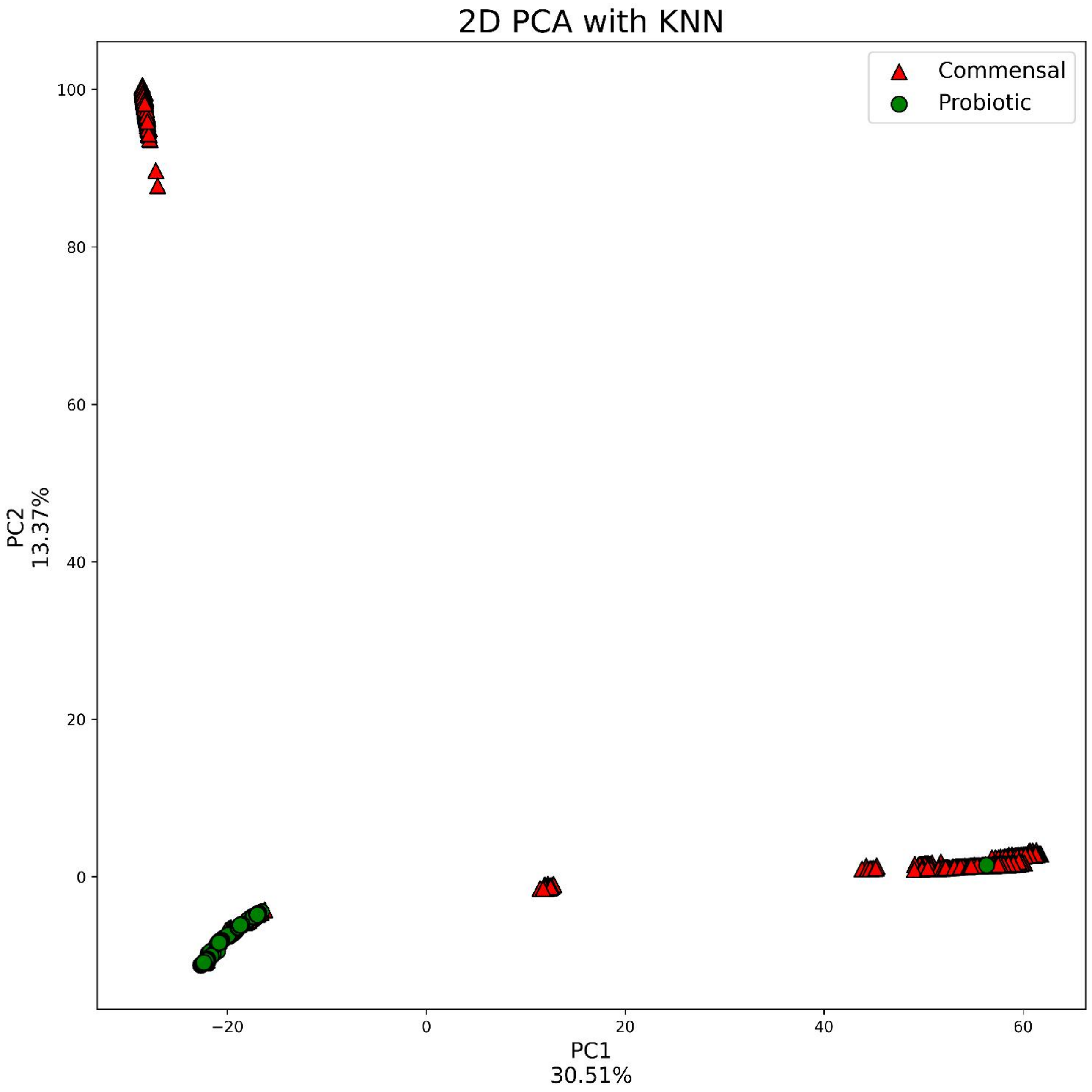

Supplementary Figure S1. Data visualization after dimensional reduction through PCA analysis.
